# Fully dissolved glucose-responsive insulin delivery system based on a self-immolative insulin prodrug and glucose oxidase

**DOI:** 10.1039/d5sc02817e

**Published:** 2025-08-11

**Authors:** Satoshi Kitaoka, Minori Kojima, Miho Koita, Hiroki Koyama, Chisato Mori, Mako Okabe, Ryusei Ando, Kaede Kobayashi, Ryo Watanabe, Yuki Takano, Tony D. James, Yuya Egawa

**Affiliations:** a Faculty of Pharmacy and Pharmaceutical Sciences, Josai University 1-1 Keyakidai, Sakado Saitama 350-0295 Japan yegawa@josai.ac.jp; b Department of Chemistry, University of Bath Bath BA2 7AY UK; c School of Chemistry and Chemical Engineering, Henan Normal University Xinxiang 453007 P. R. China

## Abstract

Among various treatment options for diabetes, insulin therapy remains an important approach, but it inevitably carries the risk of hypoglycaemia, particularly due to dosing errors or unexpected glucose fluctuations. To address this challenge, glucose-responsive insulin delivery systems that release insulin based on blood glucose levels have emerged as a promising solution. In this study, we developed a fully dissolved glucose-responsive insulin delivery system using *p*-borono-phenylmethoxycarbonyl-modified insulin aspart (BPmoc-Ins-Asp) and glucose oxidase (GOx). This system uses BPmoc-Ins-Asp as a prodrug that remains inactive until activated by hydrogen peroxide (H_2_O_2_), generated through GOx-mediated glucose oxidation. The BPmoc group undergoes a self-immolative reaction in response to H_2_O_2_, decomposing into boric acid, *p*-quinone methide, and CO_2_, thereby restoring the amino group to its original state. High-performance liquid chromatography (HPLC) confirmed the conversion of BPmoc-Ins-Asp into active Ins-Asp in the presence of GOx and glucose. Cell-based assays demonstrated that BPmoc-Ins-Asp effectively masks insulin activity until activation. Once activated, the released Ins-Asp promoted glucose transporter 4 (GLUT4) translocation, mimicking the physiological effects of insulin. *In vivo* studies further validated the system's glucose responsiveness, demonstrating glucose-lowering effects specifically under hyperglycaemic conditions, with no effect during normoglycaemic states. Unlike gel- or particle-based systems, this fully dissolved liquid formulation enables the use of thin needles for self-administration, simplifies manufacturing, and ensures consistent production quality, making it particularly suitable for clinical applications. These advantages underscore its potential for precise glucose control while minimizing the risk of hypoglycaemia.

## Abstract 2

Glucose oxidase generates hydrogen peroxide in a glucose concentration-dependent manner, cleaving the self-immolative moiety of the insulin prodrug to release the insulin analogue, enabling insulin activity only under hyperglycaemia and reducing hypoglycaemia risk.

## Graphical abstract

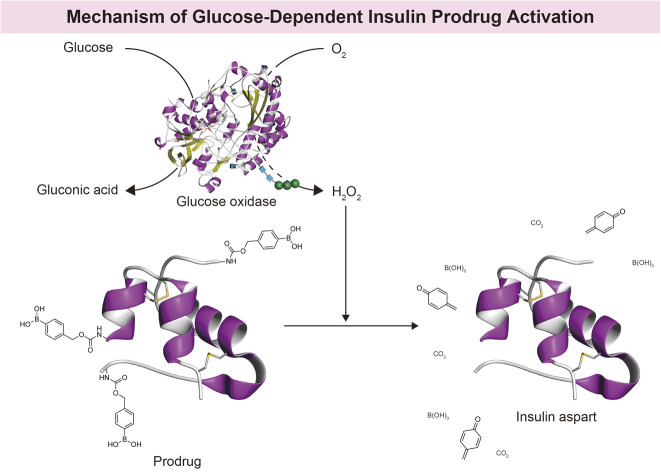

## Introduction

Insulin is the only hormone known to directly decrease blood glucose levels. It is administered to patients with type 1 diabetes who lack endogenous insulin production, and to patients with type 2 diabetes, who exhibit reduced insulin secretion or response.^1^ As a therapeutic agent, the most common and serious side effect of insulin is hypoglycaemia.^2^ Hypoglycaemia can result from inappropriate insulin dosing, such as miscalculated doses or excessive infusion rates. Other risk factors include missed meals, unadjusted insulin during vigorous exercise, and alcohol consumption on an empty stomach. Mild hypoglycaemia can cause drowsiness or dizziness, while severe hypoglycaemia can lead to cardiovascular events, coma, or even death.^3,4^

Regular blood glucose monitoring is essential for preventing hypoglycaemia and recognizing early symptoms. In recent years, continuous glucose monitoring (CGM) and flash glucose monitoring systems have been widely adopted to track glucose fluctuations, demonstrating improved glycaemic control when combined with insulin pumps.^5–7^ However, a recent study revealed that, among its participants, 25.6% of type 1 diabetes patients with CGM systems experienced at least one severe hypoglycaemic episode in the previous six months.^8^ Another study shows that, despite increasing adoption of insulin pumps and CGM, the incidence of severe hypoglycaemia remains considerable, with 7.0% of self-injectors and 5.1% of insulin pump users reporting at least one episode per year.^9^ Hypoglycaemia prevention thus remains a critical challenge.

One promising approach to reduce hypoglycaemia risk involves developing glucose-responsive insulin delivery systems.^10^ These systems regulate insulin release based on blood glucose levels, enhancing release during hyperglycaemia and reducing it during hypoglycaemia. This strategy has the potential to optimise glycaemic control and reduce the risk of hypoglycaemia, addressing a key challenge in diabetes management.

**Scheme 1:**
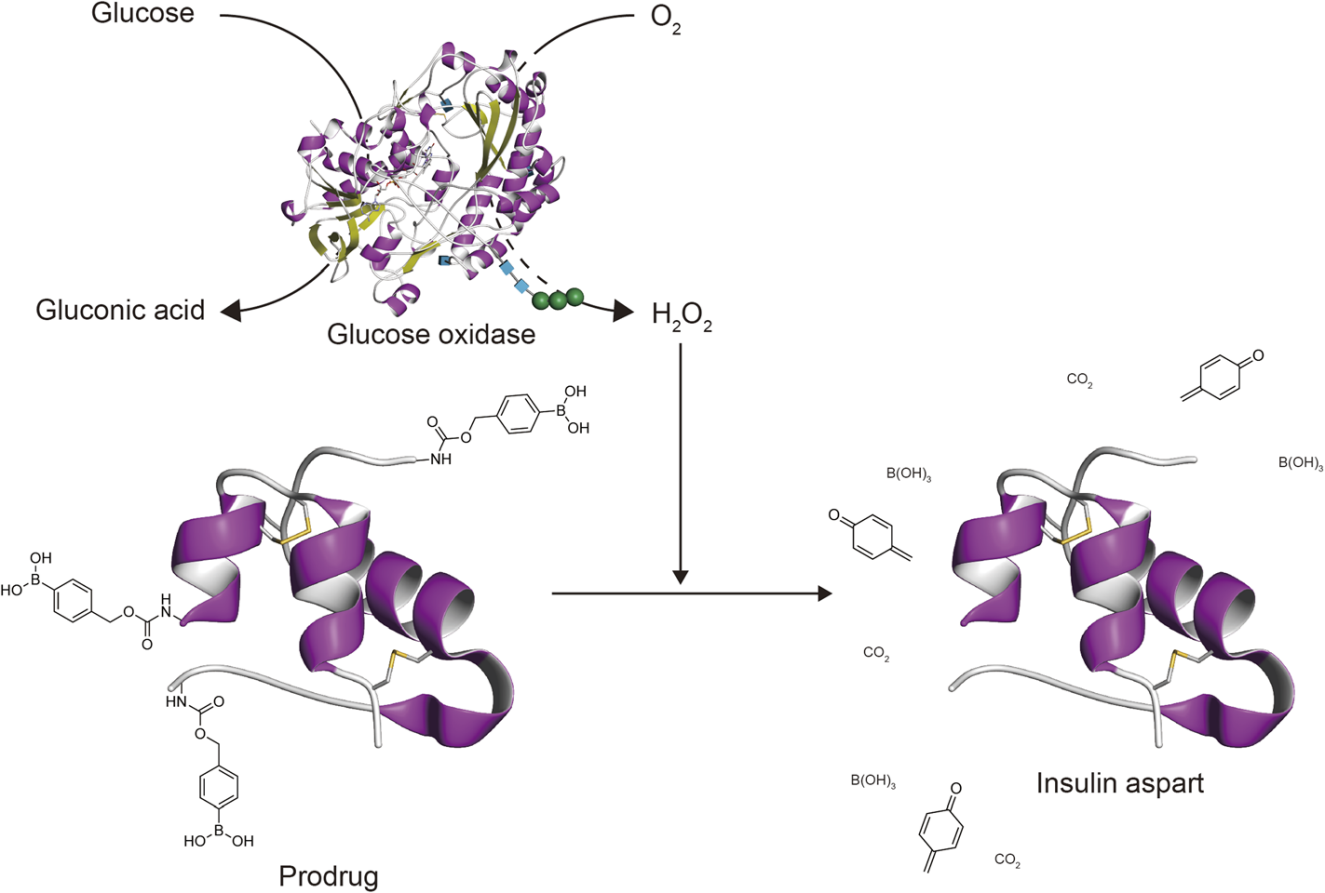
Activation of BPmoc-Ins-Asp through self-immolative elimination to produce Ins-Asp, triggered by H_2_O_2_ generated during glucose oxidation.

Among several strategies, boronic acids have been extensively studied for their glucose-responsive properties.^11–17^ Two main mechanisms have been explored: reversible covalent bond formation with polyols, including glucose, and an irreversible reaction with hydrogen peroxide (H_2_O_2_). This study focuses on the latter, where glucose oxidase (GOx) plays a key role by converting glucose concentration into H_2_O_2_ concentration, which subsequently triggers the degradation of boronic acid derivatives.^18–22^ Specifically, among the boronic acid derivatives, *p*-borono-phenylmethoxycarbonyl (BPmoc) group undergoes a reaction with H_2_O_2_, decomposing into boric acid, *p*-quinone methide, and CO_2_.^23–26^ This self-immolative reaction allows BPmoc-modified amino groups to revert to their original state upon exposure to H_2_O_2_ (Scheme 1).

Previous studies have demonstrated the feasibility of using BPmoc-modified compounds for glucose-responsive insulin delivery. Ikeda *et al.* developed hydrogels by self-assembly of BPmoc-phenylalanine dipeptide (BPmoc-FF) through π–π interactions and hydrogen bonding.^23^ These hydrogels encapsulated GOx and insulin, enabling glucose-responsive insulin release. Hu *et al.* expanded this concept by creating polymer vesicles that incorporated BPmoc groups and polyethylene glycol (PEG).^25^ These vesicles encapsulated GOx and insulin and were designed for use as microneedle patches. Their innovative designs showcased the potential of a glucose-triggered insulin delivery platform.

Despite their innovative nature, both hydrogels and particle-based formulations face practical challenges. Hydrogels are highly viscous and require high injection pressure. This makes them unsuitable for practical use with thin needles commonly employed in self-injection devices.^27,28^ Particle-based systems, such as polymer vesicles, struggle to maintain uniformity in critical parameters like particle size, drug loading amount, and glucose permeability—key factors for consistent therapeutic efficacy and glucose responsiveness. These difficulties become even more pronounced during large-scale production, limiting their viability for widespread clinical use.

To overcome the limitations of gel- and particle-based formulations, this study introduces a fully dissolved liquid formulation that leverages the superior solubility of insulin aspart (Ins-Asp) over native insulin.^29^ To achieve glucose-responsive activation, Ins-Asp was modified with BPmoc groups to create a prodrug, BPmoc-Ins-Asp, which remains inactive until activated by H_2_O_2_ generated *via* GOx-catalysed glucose oxidation. This strategy, involving a BPmoc-modified drug and GOx, was originally developed and reported by our team in a previous study.^30^ At that time, the BPmoc modification was applied to native human insulin, which has relatively low solubility. As a result, BPmoc-modified insulin displayed poor solubility, necessitating its formulation as a suspension. With this suspension formulation, the particles of BPmoc-modified insulin had to dissolve before activation, and the dissolution process led to variations in the activation time. This posed a significant challenge for accurate kinetic evaluation of the prodrug's activation process, as the dissolution step interfered with the precise measurement of activation kinetics and glucose responsiveness.

Our fully dissolved liquid-type glucose-responsive insulin formulation differs in its usage from the so-called artificial pancreas-type glucose-responsive insulin formulations. An artificial pancreas stores multiple doses of insulin and releases it in response to glucose fluctuations over a long period. Despite over 30 years of effort, the development of artificial pancreases has been hindered by technical difficulties, limiting practical implementation. Notably, administering multiple doses of insulin carries the risk of inducing hypoglycaemia, which is a significant concern.

By contrast, our fully dissolved liquid formulation is designed for single-dose administration. Since it does not require multiple insulin doses like an artificial pancreas, the risk of hypoglycaemia is minimized. This single-dose glucose-responsive formulation offers a practical approach toward the clinical implementation of glucose-responsive insulin. It has the potential to serve as a safer alternative to currently available rapid-acting insulin formulations.

The primary theoretical advantage of our system lies in its carrier-free design, which enables straightforward and precise kinetic tuning of glucose-responsive behaviour. In conventional carrier-based systems, such as those employing gels or particles, the overall response kinetics are affected by multiple factors, including the rate of glucose diffusion into the carrier and the subsequent release of insulin. As a result, simply varying the amount of GOx offers limited flexibility in modulating system responsiveness. In contrast, our carrier-free approach allows direct and efficient control of the response rate solely by adjusting the amount of GOx. This aspect is discussed in greater detail in the main text.

From a practical standpoint, the absence of carriers offers several significant benefits. In conventional carrier-based formulations, achieving uniform loading of each component onto the carrier and maintaining consistent carrier sizes—such as particle dimensions—are essential, yet they pose significant challenges for reproducible manufacturing and scale-up. In contrast, our system is a carrier-free liquid formulation, which inherently eliminates concerns related to formulation uniformity and particle size control. Furthermore, as a liquid formulation, it can be administered using a fine needle suitable for self-injection by patients, offering superior usability.

Similar to our formulation, MK-2640—developed by Merck & Co., Inc.—represents a carrier-free glucose-responsive insulin system.^31,32^ To the best of our knowledge, it remains the only glucose-responsive insulin system—excluding those involving insulin pumps—that has advanced to a Phase 1 clinical trial, underscoring the practical and clinical relevance of carrier-free formulations. MK-2640 is an insulin analogue chemically modified with mannose-based carbohydrate moieties to enable binding to mannose receptor C-type 1 (MRC1), a lectin receptor predominantly expressed on macrophages and liver Kupffer cells. MRC1 plays a key role in the innate immune system by recognizing and internalizing pathogen-associated high-mannose glycoproteins. MK-2640 was designed to exploit this clearance mechanism to achieve glucose responsiveness: under hypoglycaemic conditions, MK-2640 is rapidly removed from circulation through MRC1-mediated endocytosis, while under hyperglycaemic conditions, elevated glucose levels competitively inhibit MRC1 binding, slowing MK-2640 clearance and prolonging its glucose-lowering activity.

Although preclinical studies in dogs and minipigs demonstrated promising glucose-dependent pharmacokinetics, MK-2640 failed to show comparable efficacy in humans. This failure was attributed to multiple factors, including reduced insulin receptor binding affinity due to the carbohydrate modification—resulting in diminished insulin bioactivity—and the limited translatability of the MRC1-mediated clearance mechanism due to species-specific differences and receptor saturation. Despite encouraging preclinical data from other investigational approaches, no candidate has progressed beyond the preclinical stage, emphasizing the substantial hurdles facing clinical translation in this field. In this context, our proposal of a carrier-free glucose-responsive insulin formulation based on a fundamentally different mechanism represents a significant advance.

Unlike MK-2640, our system does not rely on receptor-mediated clearance pathways that are susceptible to species-dependent variability, thereby offering improved translational reliability. Furthermore, by employing a prodrug that is converted into pharmacologically active insulin (Ins-Asp) under glucose-responsive conditions, our approach overcomes the issue of reduced insulin potency observed in MK-2640, addressing a key limitation of previous designs.

In this study, BPmoc-Ins-Asp was successfully evaluated in its dissolved state, owing to its high solubility. The activation of BPmoc-Ins-Asp was analysed using HPLC, while the insulin activity of Ins-Asp generated from the prodrug was assessed through *in vitro* cell-based experiments. Animal studies confirmed the glucose responsiveness of the formulation *via* continuous blood glucose monitoring. Furthermore, toxicity studies were conducted to evaluate its safety. Detailed results are presented in the following sections.

## Materials and methods

### Materials

Novorapid® injection (100 units per mL, 10 mL) was purchased from Novo Nordisk Pharma Ltd (Tokyo, Japan). The injection solution was lyophilised and used as it contained 35 mg of Ins-Asp. Acetonitrile (AcCN, super-dehydrated), AcCN for high-performance liquid chromatography (HPLC), AcCN for liquid chromatography-mass spectrometry (LC-MS), ammonium hydrogen carbonate, *N*,*N*-dimethylformamide (DMF, super-dehydrated), dimethyl sulfoxide (DMSO, super-dehydrated), methanol (MeOH, for HPLC), d-glucose, 30% H_2_O_2_ aqueous solution, triethylamine, lithium hydroxide monohydrate, *N,N*′-disuccinimidyl carbonate (DSC), (+/–)-dithiothreitol (DTT), formic acid, trifluoroacetic acid (TFA) and TRITON X-100 were purchased from FUJIFILM Wako Pure Chemical Corporation (Osaka, Japan). Endoproteinase Glu-C from *Staphylococcus aureus* V8 (V8 protease), glucose oxidase (from *Aspergillus niger* Type X–S, lyophilised powder, 100 000–250 000 units per g solid without added oxygen), 4-(2-hydroxyethyl)-1-piperazineethanesulfonic acid (HEPES) and paraformaldehyde were obtained from Merck KGaA (Darmstadt, Germany). Alexa Fluor™ 647 Phalloidin, donkey anti-rabbit IgG (H + L) highly cross-adsorbed secondary antibody, Alexa Fluor™ Plus 594, fetal bovine serum (FBS) (10270-106) and GLUT4 polyclonal antibody (PA5-23052) were obtained from Thermo Fisher Scientific K.K. (Tokyo, Japan). VECTASHIELD Mounting Medium with DAPI was obtained from Vector Laboratories, Inc. (Newark, CA). Antibiotic-antimycotic mixed stock solution (100×), Dulbecco's modified Eagle medium (DMEM) (4.5 g L^−1^ glucose or no glucose) with l-glutamine, without sodium pyruvate, D-PBS(−) without Ca and Mg, 45 (w/v)%-d-(+)-glucose solution, 2.5 g l^−1^-Trypsin/1 mmol l^−1^-EDTA solution, with phenol red were obtained from NACALAI TESQUE, INC. (Kyoto, Japan). Cell Meter™ Colorimetric WST-8 Cell Quantification Kit was obtained from BLD Pharmatech Ltd (Shanghai, China). Doxorubicin hydrochloride was obtained from Apollo Scientific Ltd (Manchester, UK). Ethyl isocyanatoacetate, 4-(4,4,5,5-tetramethyl-1,3,2-dioxaborolan-2-yl)benzyl alcohol, 1,3-propanediol, and tributylamine were purchased from Tokyo Chemical Industry Co. Ltd (Tokyo, Japan). Isoflurane (Japanese Pharmacopoeia) was purchased from Pfizer Japan Inc. (Tokyo, Japan). 4-(Hydroxymethyl)phenylboronic acid was bought from Combi-Blocks Inc (San Diego, CA). All other chemicals were of reagent grade or higher and used as received.

#### Note on BPmoc-Ins-Asp concentrations

BPmoc-Ins-Asp was prepared as described in the SI and used in various experiments at nominal concentrations of 0.10 mg mL^−1^ or 0.10 μM. These concentrations were calculated based on the assumption of full modification (bearing three BPmoc groups) and 100% purity. However, elemental analysis indicated a purity of approximately 71%. Therefore, the effective concentration of BPmoc-Ins-Asp in all experiments was lower than the nominal value.

### Methods

#### Analysis of BPmoc-Ins-Asp modification positions by LC-MS

V8 protease was dissolved in ultrapure water to prepare a 2.0 mg mL^−1^ stock solution. Solutions of unmodified Ins-Asp or BPmoc-Ins-Asp (0.10 mg mL^−1^) were prepared in 10 mM ammonium hydrogen carbonate buffer (pH 8). To each 250 μL sample solution, 4.0 μL of V8 protease stock was added and incubated for 24 h at 37 °C. After incubation, DTT was added to a final concentration of 100 mM to cleave the disulphide bonds, followed by a further 1 h incubation at 37 °C. The samples were then analysed by LC-MS.

#### LC-MS analysis

The column was an XSelect CSH C18 (3.5 μm, 150 × 2.1 mm i.d., Waters, Milford, MA) and the temperature was maintained at 40 °C. The composition of mobile phase A was water/formic acid (100 : 0.1, v/v) and that of mobile phase B was MeOH/formic acid (100 : 0.1, v/v). The HPLC system (LC-20AT, SPD-20A, Shimadzu Corporation, Kyoto, Japan) was operated in binary gradient mode with a linear increase from 5 to 60% for mobile phase B over 60 min, followed by 30 min at 60% for mobile phase B and ending with a 10-min column re-equilibration with 5% mobile phase B before the next injection.

The mass spectrometer was a 4000 QTRAP (SCIEX, Framingham, MA) and detection was performed in Q3 MS mode. The ion source used was Turbo V Spray. Ionisation was performed in negative ion mode and the various other parameters were set as follows: curtain gas: 10 psi; Turbo V ionspray voltage: −4.5 kV; ion source gas 1 : 30 psi; ion source gas 2 : 80 psi; source temperature: 500 °C.

#### Cell culture

HeLa human cervical cancer cells were obtained from Riken BioResource Research Center (Ibaraki, Japan). Human keratinocyte cell line (HaCaT) was obtained from CLS Cell Lines Service GmbH (Eppelheim, Germany). Cells were cultured in DMEM supplemented with 10% heat-inactivated FBS and 1% antibiotic–antimycotic mixed stock solution at 37 °C in a humid incubator containing ambient air supplemented with 5% CO_2_.

#### Immunocytochemistry

HeLa cells were seeded in 8-well chamber slides and incubated in a CO_2_ incubator for 24 h. Prior to seeding, the cells were cultured in DMEM containing either 5 mM or 20 mM glucose for two passages to allow adaptation to the respective glucose concentrations. The medium was changed to serum-free medium and cultured for a further 24 h. Subsequently, the medium was replaced with glucose-free serum-free medium, and the cells were incubated for an additional 2 h. After this incubation, the medium was replaced with fresh DMEM containing both glucose and serum, and Ins-Asp was added to a final concentration of 0.10 μM. The conditions BPmoc-Ins-Asp (0.10 μM), BPmoc-Ins-Asp (0.10 μM) + GOx (0.10 μM) were also applied. After 20 min of addition, a 4% paraformaldehyde/PBS (−) solution was added, and the cells were fixed by incubation at room temperature for 10 min. After three washes with PBS (−), blocking buffer (3% FBS, 0.1% Triton X-100/PBS) was added for blocking and permeabilization. Primary antibody solution (anti-GLUT4 polyclonal antibody/blocking buffer) was added and incubated overnight at 4 °C. After three washes with PBS (−), secondary antibody solution was added and incubated for 1 h at room temperature in the dark. After three additional washes with PBS (−), Alexa Fluor™ 647 Phalloidin was added and incubated for 30 min at room temperature. After additional PBS washes, the cells were mounted with VECTASHIELD containing DAPI and images were captured using a confocal laser scanning microscope (FV3000, Olympus, Tokyo, Japan).

#### HPLC analysis of BPmoc-Ins-Asp degradation to Ins-Asp with GOx

Aqueous solutions (10 mL) of BPmoc-Ins-Asp (0.10 mg mL^−1^), GOx (1.0, 4.0, 10, 16 μg mL^−1^) and glucose (5.0, 20, 40, 80 mM) were prepared using HEPES buffer (10 mM, pH 7.4) and incubated at 37 °C. The amount of Ins-Asp produced was analysed by HPLC. At the scheduled time, 1.0 mL of the reaction solution was sampled and mixed with 2.0 mL of mobile phase A for HPLC to stop the BPmoc group elimination reaction and analysed by HPLC.

HPLC analysis was conducted using an octadecylsilyl (ODS) column (5 μm, 150 × 4.6 mm i.d., CAPCELL PAK C18, Shiseido Fine Chemicals, Tokyo, Japan) and gradient elution at a flow rate of 1.0 mL min^−1^. Mobile phase A consisted of AcCN/water/TFA (300 : 700 : 1, v/v/v) and mobile phase B consisted of AcCN/water/TFA (950 : 50 : 1, v/v/v). The HPLC system (LC-20AT, SPD-20A, Shimadzu Corporation, Kyoto, Japan) was operated in gradient mode with a linear increase from 0 to 33.3% for mobile phase B over 20 min. Elution was monitored by absorbance at 271 nm.

#### Animal handling

Seven-week-old male Wistar and GK rats were purchased from Japan SLC, Ltd (Shizuoka, Japan). Animals were housed in a facility maintained at 24 ± 1 °C with 55 ± 5% humidity and a 12-h light/dark cycle (08 : 00 to 20 : 00). All animal procedures were performed in accordance with the Guidelines for Care and Use of Laboratory Animals of Josai University and approved by the Institutional Animal Care and Use Committee of Josai University (Approval No. JU 22079 and JU 23071).

#### Treatment protocols

Blood glucose levels were measured using a continuous glucose monitoring system (FreeStyle Libre system, Abbott Japan Co., Tokyo, Japan). The day before the measurement, the sensor was attached to the back of the rats under isoflurane anaesthesia. The sensor was sutured to the rat's skin at multiple points along its edges to ensure secure attachment.

Saline was used to prepare the injection formulations: BPmoc-Ins-Asp (0.10 mg mL^−1^), BPmoc-Ins-Asp (0.10 mg mL^−1^) + GOx (4.0 μg mL^−1^) and Ins-Asp (0.10 mg mL^−1^). These formulations were administered to rats restrained in a Ballman cage (s.c., 1.0 mL kg^−1^ body weight).

Water for injection was used to prepare the glucose solution (0.20 g mL^−1^) for the oral glucose tolerance test (OGTT). The rats were fasted for 16 h, after which the glucose solution (p.o., 10 mL kg^−1^ body weight) was administered.^33^

#### Haematoxylin–eosin staining

Each formulation, GOx (4.0 μg mL^−1^), BPmoc-Ins-Asp (0.10 mg mL^−1^), BPmoc-Ins-Asp (0.10 mg mL^−1^) + GOx (4.0 μg mL^−1^) and Ins-Asp (0.10 mg mL^−1^), was administered to Wistar rats in the same area once daily for 5 days. Two hours after the last administration, the subcutaneous tissue at the site of administration was excised by punch biopsy under isoflurane anaesthesia. After excision, the tissue was fixed with 10% neutral buffered formalin. The tissue was then embedded in paraffin and sectioned. The sections were stained with haematoxylin and eosin (HE). Detection was performed with a microscope (BZ-X810, KEYENCE, Osaka, Japan).

#### Cell proliferation assay

The effect of the formulation on cell proliferation was assessed using a WST-8 assay. HaCaT cells were seeded in 96-well plates at a density of 5 × 10^3^ cells per well and incubated in a CO_2_ incubator for 24 h. Different test samples (GOx (25 ng mL^−1^), BPmoc-Ins-Asp (0.10 μM), BPmoc-Ins-Asp (0.10 μM) + GOx (25 ng mL^−1^), Ins-Asp (0.10 μM), BPmoc-modified glycine (BPmoc-Gly) (0.10 μM), BPmoc-Gly (0.10 μM) + GOx (25 ng mL^−1^), doxorubicin (low: 0.10 μM; high: 1.0 μM)) were prepared and added to the cells and incubated for 24 h. After 48 h, WST-8 reagent was added. The absorbance at 1 h was then measured in a Cell Imaging Multi-Mode Reader (Cytation 5, Agilent BioTek, Santa Clara, CA).

#### Statistical analysis and protein visualization

Experimental values are expressed as the mean ± standard deviation (SD). Significant differences were calculated using one-way ANOVA, followed by Tukey's multiple comparison test. These calculations were performed using GraphPad Prism (version 9.3.1) statistical analysis software.

The area under the curve from 0 to 2 h (AUC_0–2_) of blood glucose levels was calculated using pharmacokinetic analysis software, Numeric Analysis Program for Pharmacokinetics (Napp) (version 3.071β).^34^

The protein structures in Scheme 1 and the Table of Contents figure were visualized using protein structure data from the PDBj database *via* the molecular viewer Molmil.^35^ The data for glucose oxidase from *Aspergillus niger* (PDB ID: 1CF3) was used as is,^36^ while BPmoc-Ins-Asp and Ins-Asp was visualized using human insulin (PDB ID: 3I3Z).^37^

## Results and discussion

### Synthesis of BPmoc-Ins-Asp and BPmoc-Gly

BPmoc-modified *N*-hydroxysuccinimide (BPmoc-NHS) was synthesized according to a previously reported method.^30^ Briefly, 4-(hydroxymethyl)phenylboronic acid was protected with 1,3-propanediol to enhance solubility, and it was reacted with *N,N*′-disuccinimidyl carbonate (DSC) to give BPmoc-NHS as an active ester. BPmoc-NHS was reacted with lyophilised Ins-Asp from a commercial solution prior to modification. BPmoc-Ins-Asp was purified by dialysis against water, lyophilised, and stored at −20 °C. The purity of the obtained BPmoc-Ins-Asp was evaluated by elemental analysis, assuming that impurities do not contain nitrogen. Based on the calculated theoretical nitrogen content of 14.32% for the fully modified BPmoc-Ins-Asp (bearing three BPmoc groups), and the experimentally observed nitrogen content of 10.21%, the purity was estimated to be 71%. The concentration of BPmoc-Ins-Asp used in experiments was calculated assuming full modification and 100% purity; therefore, the actual effective concentration is lower than the nominal value.

BPmoc-modified glycine (BPmoc-Gly) used in a toxicity study was prepared by reacting the pinacol ester of 4-(hydroxymethyl)phenylboronic acid with ethyl isocyanatoacetate to form a urethane bond, which was then hydrolysed with LiOH.

Detailed synthetic procedures and synthetic schemes (Scheme S1 and S2) are given in the SI.

### Fragment analysis of BPmoc-Ins-Asp by LC-MS

To determine the number and position of BPmoc modifications on Ins-Asp, BPmoc-Ins-Asp was enzymatically degraded into fragments and analysed using LC-MS. The V8 protease from *Staphylococcus aureus* specifically cleaves the carboxyl side of glutamate residues under conditions of 10 mM ammonium hydrogen carbonate.^38^

**Fig. 1:**
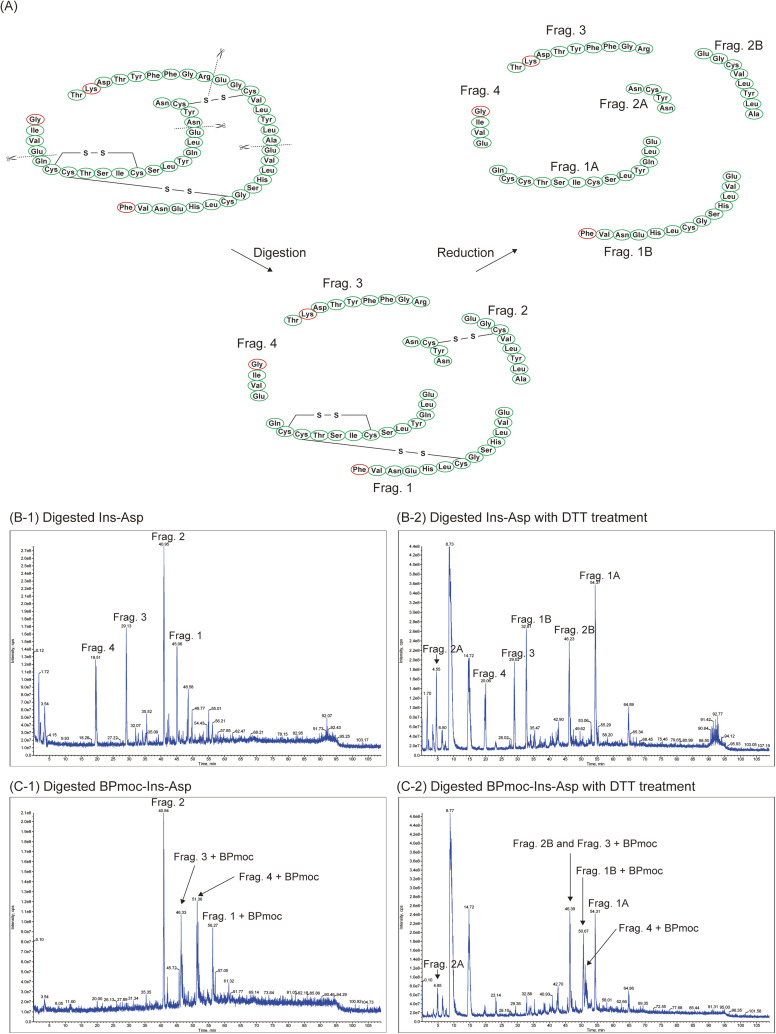
Structural analysis of Ins-Asp and BPmoc-Ins-Asp by LC-MS. (A) Amino acid sequence of Ins-Asp, its form after digestion with V8 protease, and the product further reduced with DTT. (B) LC-MS chromatograms of insulin derivatives digested with V8 protease: (B-1) digested Ins-Asp, (B-2) digested Ins-Asp with DTT treatment, (C-1) digested BPmoc-Ins-Asp, (C-2) digested BPmoc-Ins-Asp with DTT treatment.

Fig. 1(A) illustrates the structure of Ins-Asp and its expected fragments 1–4 (Frag. 1–4) upon digestion with V8 protease and subsequent reduction with DTT.

Fig. 1(B-1) shows the LC-MS chromatogram of Ins-Asp digested with V8 protease, revealing four major peaks. Based on comparison with a previous study, these peaks were identified as Frag. 1 to 4.^39^ The MS spectra of each peak are presented in Fig. S1, and the theoretical and observed *m/z* values are summarised in Table S1.

Fig. 1(C-1) shows the chromatogram of BPmoc-Ins-Asp digested in a similar manner, where the peak corresponding to Frag. 2 of Ins-Asp was found at 41 min. Meanwhile, Frag. 1, Frag. 3, and Frag. 4 were not detected, suggesting complete chemical modification of these three fragments. Three new peaks were observed in Fig. 1(C-1), and the presence or absence of BPmoc modification was investigated from the MS spectra of each peak (Fig. S3 and Table S3). It should be noted that previous studies have shown that MS spectra of compounds containing phenylboronic acid typically exhibit characteristic dehydrated ions rather than the intact molecular weight.^40^ In Fig. 1(C-1), the peaks detected at 46, 51, and 56 min included *m/z* values that were close to theoretical *m/z* values for the expected ions (Fig. S3 and Table S3). The peak at 46 min corresponded to [M_Frag. 3+BPmoc_–2H_2_O–2H]^2–^, with a theoretical *m/z* of 637.10 and an observed *m/z* of 638.00. Similarly, the peak at 51 min corresponded to [M_Frag. 4+BPmoc_–H_2_O–H]^–^, with a theoretical *m/z* of 575.43 and an observed *m/z* of 576.40. The peak at 56 min corresponded to [M_Frag. 1+BPmoc_–H_2_O–2H]^2–^, with a theoretical *m/z* of 1564.67 and an observed *m/z* of 1564.10. These results showed that each BPmoc group was incorporated as a single modification into Frag. 3, Frag. 4, and Frag. 1.

**Fig. 2:**
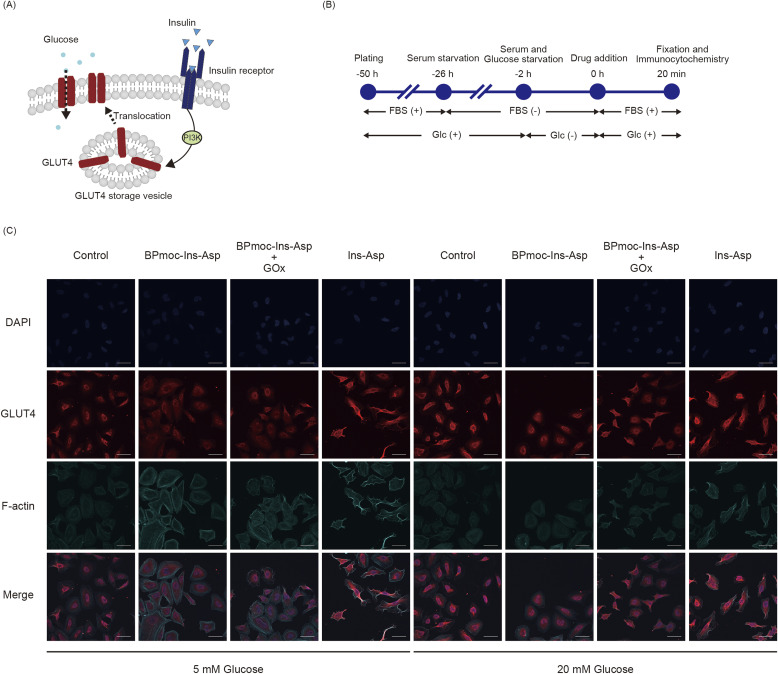
Investigation of insulin activity with GLUT4 translocation to the plasma membrane. (A) Schematic of GLUT4 translocation to the plasma membrane after insulin signalling, (B) the experimental time course, (C) localisation of intracellular GLUT4 by confocal laser scanning microscopy after immunostaining (blue, DAPI; red, GLUT4; Cyan, F-actin; scale bar, 50 μm).

To further investigate the position of the BPmoc group in Frag.1, DTT was used to cleave the disulphide bond. In Fig. 1(B-2) for Ins-Asp reduced with DTT, Frag. 1A and Frag. 1B were observed (Fig. S2 and Table S2). In Fig. 1(C-2) for DTT-reduced BPmoc-Ins-Asp, Frag. 1B was not present, but a new peak was seen at 50.67 min. The MS spectrum from this peak included an *m/z* value of 812.00, which was close to the expected *m/z* of 811.32 for [M_Frag. 1B+BPmoc_–2H_2_O–2H]^2−^, indicating that Frag. 1B was modified (Fig. S4 and Table S4).

In summary, BPmoc groups were introduced at three specific sites: Frag. 1B, Frag. 3, and Frag. 4 of Ins-Asp. Each fragment contains a single amino group: Frag. 1B features an amino group at the N-terminus of the insulin B chain, Frag. 3 has one at the 29th lysine residue of the B chain, and Frag. 4 has one at the N-terminus of the insulin A chain. These results strongly suggest that BPmoc groups were specifically introduced at these amino groups.

It has been reported that acylation of the N-terminus of Gly of the A-chain markedly reduces insulin activity.^41^ Furthermore, acylation at the N-terminus of Phe of the B-chain has also been shown to result in insufficient insulin activity.^42^ In the case of the BPmoc-Ins-Asp obtained in this study, chemical modifications are introduced at both of these N-terminal residues, and this is considered to be the main factor contributing to the loss of insulin activity.

### Evaluation of prodrug conversion into Ins-Asp using cultured cells

In our system, the BPmoc group should mask the pharmacological activity of Ins-Asp and its removal is essential to restore insulin activity. To confirm the absence or presence of insulin activity, the localisation of glucose transporter 4 (GLUT4) was examined by immunostaining. Insulin binds to and activates the insulin receptor, a tyrosine kinase that triggers a signalling cascade. This activation initiates the phosphatidylinositol 3-kinase (PI3K)/AKT pathway, which facilitates the translocation of GLUT4 to the plasma membrane, thereby promoting glucose uptake into the cell (Fig. 2(A)).^43^ To investigate GLUT4 translocation, this study used 0.10 μM of the insulin derivative, as in a previous study.^44^ Two glucose concentrations were set in the culture medium: 5 mM as a low-glucose (hypoglycaemic) condition and 20 mM as a high-glucose (hyperglycaemic) condition. Specimens were prepared according to the time course shown in Fig. 2(B) and analysed by confocal laser scanning microscopy (Fig. 2(C)).

F-actin, shown in cyan, was used as a counterstain to clearly delineate the cell outline and to provide spatial context for assessing the intracellular localization of GLUT4. Moreover, since GLUT4 translocates to the plasma membrane along F-actin filaments, co-staining of GLUT4 and F-actin enabled a more detailed evaluation of the pharmacological effect of insulin.^45^

Under the 5 mM glucose condition, GLUT4 translocation to the plasma membrane was detected exclusively in cells treated with Ins-Asp. In these cells, the red fluorescence signal derived from GLUT4 was distributed along intracellular F-actin filaments and extended to the cell periphery, indicating successful translocation. In contrast, cells treated with BPmoc-Ins-Asp alone or in combination with GOx showed cytoplasmic retention of the GLUT4 signal, similar to untreated controls. Notably, strong punctate red signals were observed in the perinuclear region, likely representing vesicle-trapped GLUT4. These results demonstrate that under low-glucose conditions (5 mM), BPmoc-Ins-Asp did not exert insulin activity, regardless of GOx co-treatment.

Under the 20 mM glucose condition, cells treated with either Ins-Asp or BPmoc-Ins-Asp in combination with GOx exhibited a diffuse red GLUT4 signal throughout the cytoplasm, outlining the cell shape, consistent with plasma membrane localisation. In contrast, the control and BPmoc-Ins-Asp alone groups displayed centrally clustered GLUT4 signals with poorly defined cell boundaries. Without the F-actin (cyan) counterstain, it was difficult to identify the plasma membrane in these groups. Thus, under high-glucose conditions, BPmoc-Ins-Asp elicited insulin-like activity only in the presence of GOx. Importantly, BPmoc-Ins-Asp alone did not induce GLUT4 translocation, even under hyperglycaemic conditions.

Collectively, these findings validate the functionality of our glucose-responsive activation system: the insulin activity of Ins-Asp is masked by BPmoc conjugation and reactivated in the presence of GOx and 20 mM glucose. Moreover, the lack of activity under 5 mM glucose further highlights the glucose-dependent nature of this mechanism.

### Kinetics of activation of BPmoc-Ins-Asp to Ins-Asp

For clinical application, it is essential that the prodrug, BPmoc-Ins-Asp, respond effectively to physiological glucose concentrations. According to blood glucose criteria, the American Diabetes Association (ADA) defines diabetes mellitus as a fasting plasma glucose (FPG) level ≥126 mg dL^−1^ (7.0 mM) after at least 8 hours of fasting, a 2-hour plasma glucose level ≥200 mg dL^−1^ (11.1 mM) during an oral glucose tolerance test (OGTT), or a random plasma glucose level ≥200 mg dL^−1^ (11.1 mM) in individuals with classic symptoms of diabetes.^46^ Thus, it is critical to design prodrugs that can be reliably activated at glucose concentrations of approximately 10 mM. With this in mind, we optimised the formulation of prodrugs with a focus on their kinetic properties.

First, we observed the conversion of BPmoc-Ins-Asp to Ins-Asp in the presence of H_2_O_2_. In the HPLC chromatogram, BPmoc-Ins-Asp appeared as a single peak at 12 min (Fig. S5(A)). Upon addition of H_2_O_2_, a new peak corresponding to Ins-Asp appeared at 8 min (Fig. S5(B)), and its intensity increased with higher H_2_O_2_ concentrations (Fig. S5(C)). At an H_2_O_2_ concentration of 5.0 mM, the amount of Ins-Asp produced reached a plateau after 30 min, indicating that the conversion was complete. These results confirm the H_2_O_2_ responsiveness of BPmoc-Ins-Asp.

**Fig. 3:**
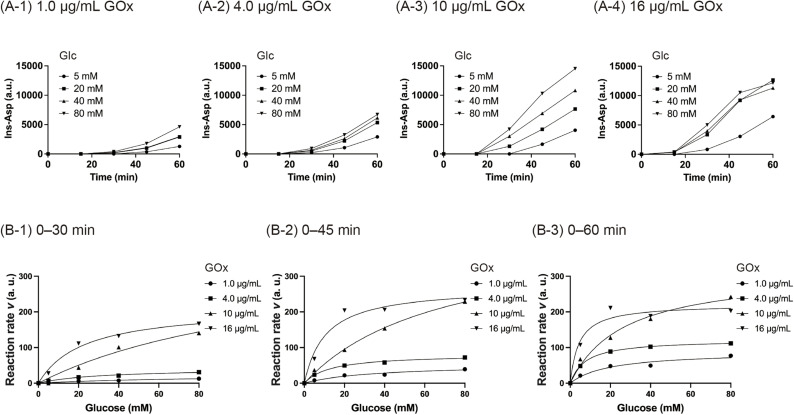
Kinetics studies of Ins-Asp formation by prodrug activation. (A) Ins-Asp production profiles from BPmoc-Ins-Asp (0.10 mg mL^−1^) mixed with GOx (A-1: 1.0 μg mL^−1^, A-2: 4.0 μg mL^−1^, A-3: 10 μg mL^−1^, A-4: 16 μg mL^−1^) at different glucose concentrations (5.0, 20, 40, and 80 mM). (B) Average reaction rates at various glucose concentrations over different time intervals (B-1: 0–30 min, B-2: 0–45 min, B-3: 0–60 min).

The next step was to investigate the effect of GOx concentration on the activation of BPmoc-Ins-Asp. The concentration of BPmoc-Ins-Asp was kept constant at 0.10 mg mL^−1^, while the GOx concentration (1.0, 4.0, 10 and 16 μg mL^−1^) and the glucose concentration (5.0, 20, 40 and 80 mM) were varied. Under all conditions, the conversion to Ins-Asp increased with increasing glucose concentration (Fig. 3(A)), confirming that the proposed glucose-responsive mechanism was functional. Furthermore, the results show that the activation rate of BPmoc-Ins-Asp could be modulated by adjusting the concentration of GOx.

The average reaction rate was calculated by dividing the peak area at each time point by the corresponding time period, and then plotted against the glucose concentration (Fig. 3(B)). The graph revealed that under the condition of a GOx concentration of 16 μg mL^−1^, the reaction rate increased rapidly even when the glucose concentration was below 10 mM. This suggests that at 16 μg mL^−1^ GOx, BPmoc-Ins-Asp is converted to Ins-Asp even at normal blood glucose levels, potentially causing hypoglycaemia. In contrast, at GOx concentrations of 1.0 to 10 μg mL^−1^, the reaction rate increased gradually within the glucose concentration range of 0–10 mM. Based on these results, GOx concentrations below 16 μg mL^−1^ were considered suitable for injectable solutions.

### Pharmacological evaluation of formulation containing BPmoc-Ins-Asp and GOx

**Fig. 4:**
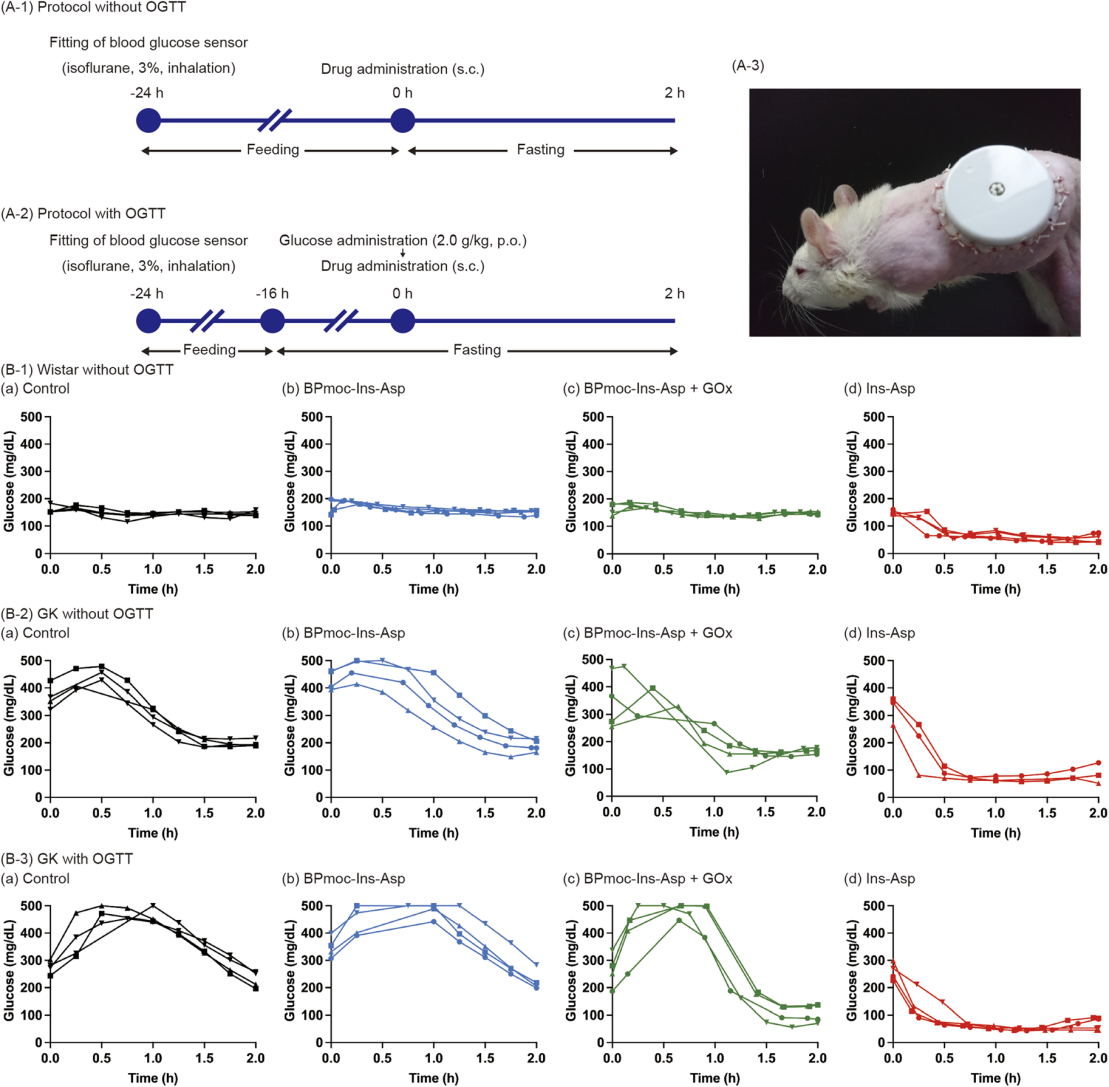
Glucose monitoring after administration of each formulation: (a) control (saline), (b) BPmoc-Ins-Asp, (c) BPmoc-Ins-Asp + GOx, (d) Ins-Asp. (A) protocols for administration: (A-1) protocol without OGTT, (A-2) protocol with OGTT, (B) glucose profiles with four formulations: (B-1) Wistar rats without OGTT, (B-2) GK rats without OGTT, (B-3) GK rats with OGTT.

The *in vivo* efficacy and appropriate dosage of BPmoc-Ins-Asp and GOx formulations were evaluated based on *in vitro* kinetic studies. Considering efficacy and dosage (1.0 mL kg^−1^), the concentration of BPmoc-Ins-Asp was fixed at 0.10 mg mL^−1^. GOx was set at 10 μg mL^−1^ and 4.0 μg mL^−1^ because *in vitro* kinetic results suggested that the appropriate GOx concentration was below 16 μg mL^−1^. Wistar rats were treated according to the protocol shown in Fig. 4(A-1), and their glucose levels were monitored with an attached glucose monitoring system (Fig. 4(A-3)).

The group receiving the formulation (0.10 mg mL^−1^ BPmoc-Ins-Asp + 10 μg mL^−1^ GOx) tended to have lower blood glucose levels than the saline-treated control group (Fig. S6(A)). The AUC_0–2h_ of this group was significantly lower than that of the control group (Fig. S6(B)). This result is meaningful in the context that BPmoc-Ins-Asp can be activated to Ins-Asp in the body. However, it is not consistent with our concept of a glucose-responsive formulation that does not lower blood glucose levels under normoglycaemic conditions. The group treated with the formulation (0.10 mg mL^−1^ BPmoc-Ins-Asp + 4.0 μg mL^−1^ GOx) did not reduce blood glucose levels in normoglycaemic rats, and the AUC_0–2h_ was not significantly different from that of the control group (Fig. S6(B)). Thus, it was decided to further investigate the potential of this formulation.

Wistar rats with normoglycaemia were treated according to the protocol shown in Fig. 4(A-1), and four formulations were administered for evaluation: (a) control (saline), (b) 0.10 mg mL^−1^ BPmoc-Ins-Asp, (c) 0.10 mg mL^−1^ BPmoc-Ins-Asp + 4.0 μg mL^−1^ GOx, and (d) 0.10 mg mL^−1^ Ins-Asp. In the control group, blood glucose levels remained stable at approximately 150 mg dL^−1^ (8.3 mM) for 2 h (Fig. 4(B-1)(a)). Similar results were observed in the BPmoc-Ins-Asp group (Fig. 4(B-1)(b)), consistent with *in vitro* findings indicating that the insulin activity of BPmoc-Ins-Asp was masked. The data shown in Fig. 4(B-1)(c) is the same as the 0.10 mg mL^−1^ BPmoc-Ins-Asp + 4.0 μg mL^−1^ GOx data in Fig. S6(A) described previously, and it did not reduce blood glucose levels. The Ins-Asp-treated group exhibited a rapid drop in blood glucose levels, resulting in hypoglycaemia (Fig. 4(B-1)(d)); hypoglycaemia in rats is reported as a blood glucose level below 3.5 mM.^47^

For conditions with high blood glucose levels, Goto-Kakizaki (GK) rats were used, which are known as a model of type 2 diabetes.^48^ In the control group with GK rats (Fig. 4(B-2)(a)), blood glucose levels were high, ranging from 300 to 500 mg dL^−1^ (approximately 17–28 mM). Subsequently, blood glucose levels gradually decreased. This slow decline is presumed to result from endogenous insulin secretion, as GK rats partially respond to hyperglycaemia by producing their own insulin.^49^ The glucose profile of the BPmoc-Ins-Asp group was similar to that of the control group (Fig. 4(B-2)(b)). Notably, the BPmoc-Ins-Asp + GOx group showed a trend towards lower blood glucose (Fig. 4(B-2)(c)).

To simulate an increase in postprandial blood glucose levels, the formulations were administered to GK rats immediately after OGTT, according to the protocol shown in Fig. 4(A-2). In the control group, blood glucose levels peaked approximately 1 h later, followed by a gradual decline, reflecting the endogenous insulin activity of GK rats (Fig. 4(B-3)(a)). The BPmoc-Ins-Asp group showed a similar glucose profile (Fig. 4(B-3)(b)). In contrast, the BPmoc-Ins-Asp + GOx group showed a clear glucose-lowering effect, although there was a time lag (Fig. 4(B-3)(c)).

The absence of a glucose-lowering effect in normoglycemic rats is likely due to limited conversion of the prodrug to Ins-Asp under low glucose conditions. This is consistent with our *in vitro* cell-based assays, which demonstrated a marked difference in prodrug activation between 5 mM and 20 mM glucose (Fig. 2).

**Fig. 5:**
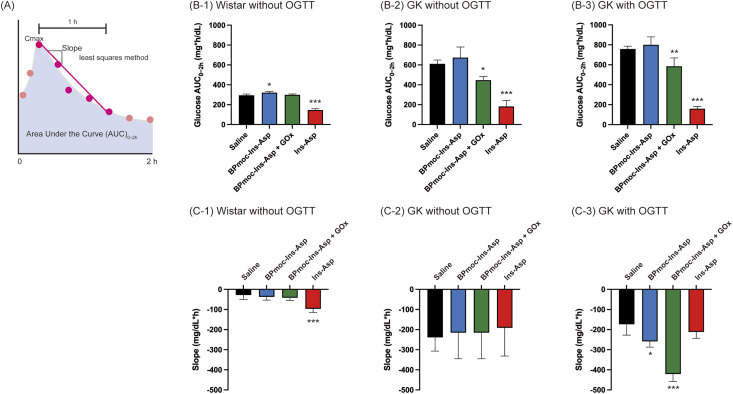
Analysis of changes in blood glucose levels. (A) Conceptual diagram of the calculated AUC_0–2h_ of the blood glucose time curve and the slope obtained by dividing the change in concentration from *t*_max_ to 1 h by 1 h (B) Glucose AUC_0–2h_ for (B-1) Wistar rats without OGTT, (B-2) GK rats without OGTT, (B-3) GK rats with OGTT. (C) Slope for (C-1) Wistar rats without OGTT, (C-2) GK rats without OGTT, (C-3) GK rats with OGTT. The results are presented as mean ± SD. Significant differences between groups were calculated using one-way ANOVA and Tukey's multiple comparison test (*vs.* control: *p < 0.05, **p < 0.01, ***p < 0.001).

To further compare the above results, AUC_0–2h_ was calculated, as conceptualised in Fig. 5(A). In Fig. 5(B-1), which shows AUC_0–2h_ in normal Wistar rats, only the Ins-Asp group showed a significant decrease compared to the control group (***p < 0.001). The BPmoc-Ins-Asp group was also significantly different from the control group; however, this was a slight increase rather than a decrease (*p < 0.05). Fig. 5 (B-2) shows the AUC_0–2h_ in GK rats without OGTT, where the BPmoc-Ins-Asp + GOx group showed a significant reduction compared to the control group (*p < 0.05). Fig. 5(B-3) shows the AUC_0–2h_ in GK rats with OGTT, in which the difference between the control and BPmoc-Ins-Asp + GOx groups was more pronounced (**p < 0.01).

As schematically shown in Fig. 5(A), the rate of blood glucose lowering was calculated as the slope from the maximum blood glucose level (*t*_max_) to 1 h later. In both normoglycaemic Wistar rats and GK rats without OGTT, the BPmoc-Ins-Asp + GOx group did not differ from the control group (Fig. 5(C-1 and 2)). However, in the GK rat study with OGTT (Fig. 5(C-3)), the BPmoc-Ins-Asp + GOx group had a significantly greater negative slope than the control group (***p < 0.001).

The comparison of blood glucose levels, AUC_0–2h_, and the rate of blood glucose reduction demonstrated that the combination of BPmoc-Ins-Asp and GOx effectively reduced blood glucose levels exclusively under hyperglycaemic conditions, without affecting normoglycaemic levels. These results confirm that our concept is functional; however, the glucose-lowering effect became evident only after 1.5 h, and a faster response is desirable in the future.

In this study, HPLC analysis and animal experiments revealed that BPmoc-Ins-Asp was converted to Ins-Asp under normoglycaemic conditions when the GOx concentration was high. From a clinical perspective, the potential risk of hypoglycaemia due to excessive GOx levels must be carefully considered. While the amount of H_2_O_2_ generated can be regulated by adjusting the GOx concentration, this alone is insufficient to achieve ideal glucose dependency. Therefore, to restrict insulin activation to hyperglycaemic conditions, it is necessary not only to optimize the GOx concentration but also to consider other contributing factors. In particular, adjusting the intrinsic glucose reactivity of GOx—through the selection of alternative isoforms or the application of protein engineering techniques—may represent a promising future approach.

### Assessment of toxicity of prodrug formulation

Because of concerns about the toxicity of the H_2_O_2_ produced by GOx and degradation products from the BPmoc group (boric acid, quinone methide), a histological examination was performed to assess potential tissue toxicity at the site of administration. While quinone methide is an active species and can react with glutathione (GSH), a scavenger of reactive oxygen species, potentially increasing oxidative stress due to GSH depletion,^50^ the levels produced under the tested conditions appeared to have no significant adverse effects.

**Fig. 6:**
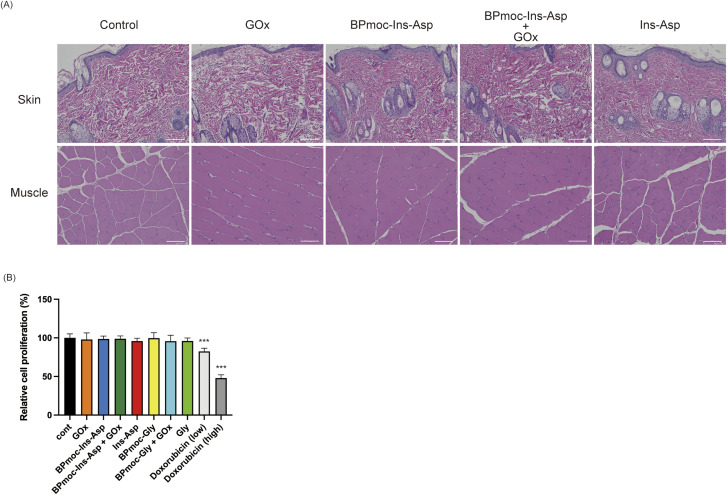
Toxicity assessment of formulation-derived compounds. (A) Haematoxylin and eosin (HE)-stained subcutaneous tissue sections from Wistar rats treated with each formulation (control: saline, GOx, BPmoc-Ins-Asp, BPmoc-Ins-Asp + GOx, Ins-Asp; subcutaneous injection, 1.0 mL kg^−1^ body weight; scale bar: 100 μm). (B) Cell proliferation assay of HaCaT cells treated with various test samples (control, GOx, BPmoc-Ins-Asp, BPmoc-Ins-Asp + GOx, Ins-Asp, BPmoc-Gly, BPmoc-Gly + GOx, Gly, and doxorubicin (low: 0.10 μM, high: 1.0 μM)) after 48 hours, using the WST-8 reagent (mean ± SD). Significant differences were analysed by one-way ANOVA and Tukey's multiple comparison tests, compared to the control (***p < 0.001).

Four injection solutions, (a) control (saline), (b) 0.10 mg mL^−1^ BPmoc-Ins-Asp, (c) 0.10 mg mL^−1^ BPmoc-Ins-Asp + 4.0 μg mL^−1^ GOx, and (d) 0.10 mg mL^−1^ Ins-Asp, were administered to Wistar rats daily for 5 days. Skin and muscle samples from the injection site were collected 2 h post-final administration and stained with haematoxylin and eosin (HE). Histological evaluation revealed no signs of inflammation, necrosis, or other structural abnormalities in skin or muscle tissue compared to the control group, as shown in Fig. 6(A).

To evaluate toxicity in *in vitro* systems, BPmoc-Gly, a glycine derivative modified with a BPmoc group, was synthesised to address the possibility that the Ins-Asp produced during prodrug activation could mask the potential toxic effects. Since glycine is present in the culture medium at a concentration of 400 μM, the additional glycine released by BPmoc-Gly activation (0.10 μM) was considered negligible. Analysis of the reaction of BPmoc-Gly with H_2_O_2_ using FAB-MS confirmed the production of glycine ([M+H]^+^ = 76) and quinone methide ([M+H]^+^ = 107), as evidenced by peaks corresponding to these masses in Fig. S7.

The toxicity associated with the activation of the prodrug formulation was evaluated using a WST-8 assay system with HaCaT cells, a type of keratinocyte.^51,52^ The results in Fig. 6(B) showed that doxorubicin, used as a positive control, inhibited cell proliferation in a concentration-dependent manner. In contrast, no significant differences in proliferation were observed in HaCaT cells treated with the prodrug formulation or other associated compounds.^53^

Taken together, the histological analysis and *in vitro* assays indicate that the formulation containing BPmoc-Ins-Asp and GOx, as well as its degradation products, does not induce significant toxicity, supporting its potential for safe therapeutic applications.

Although this study was limited to short-term evaluations, no signs of toxicity were observed within the experimental timeframe. However, comprehensive long-term safety assessments will be essential to support clinical translation. While GOx is commonly used in glucose-responsive insulin systems, its toxicological profile remains largely uncharacterized, making this study a notable contribution in that regard. Given that GOx is a non-human protein, potential immunogenicity—including allergenicity—should be carefully assessed. If immunological responses are detected, strategies such as PEGylation may be necessary to mitigate immunoreactivity. Furthermore, long-term safety concerns associated with the generation of quinone methide intermediates and the presence of boronic acid derivatives, including their potential for cumulative exposure, should be addressed in future studies.

## Conclusion

This study successfully developed and evaluated a fully dissolved, glucose-responsive insulin prodrug formulation using BPmoc-Ins-Asp and GOx. BPmoc-Ins-Asp demonstrated high solubility, allowing for precise kinetic analysis, overcoming the limitations of previous suspension-based formulations.^30^*In vitro* studies confirmed that BPmoc modification masked insulin activity, with activation occurring *via* GOx-mediated H_2_O_2_ production. *In vivo* experiments showed that BPmoc-Ins-Asp + GOx selectively lowered blood glucose levels under hyperglycaemic conditions without inducing hypoglycaemia in normoglycaemic rats, demonstrating effective glucose responsiveness.

Furthermore, histological and cell proliferation assays indicated no significant toxicity from BPmoc-Ins-Asp, GOx, or their degradation products, supporting the formulation's safety. While the formulation successfully functioned as designed, activation kinetics require further optimisation to achieve a faster glucose-lowering effect comparable to rapid-acting insulin analogues.

Overall, this study highlights the potential of a single-dose, glucose-responsive insulin formulation as a safer alternative to conventional insulin therapies. By improving activation kinetics, this approach could offer a practical solution for reducing the risk of insulin-induced hypoglycaemia, advancing diabetes management.

## Author contributions

Satoshi Kitaoka: data curation, formal analysis, investigation, methodology, visualization, and writing – original draft. Minori Kojima, Miho Koita, Hiroki Koyama, Chisato Mori, Mako Okabe, Ryusei Ando, Kaede Kobayashi, Ryo Watanabe, and Yuki Takano: investigation. Tony D. James: supervision and writing – review & editing. Yuya Egawa: conceptualization, data curation, investigation, methodology, project administration, supervision, Funding acquisition, writing – original draft, and writing – review & editing.

## Conflicts of interest

There are no conflicts to declare.

## Supplementary Material

SC-OLF-D5SC02817E-s001

## Data Availability

The data that support the findings of this study are available in the SI of this article. Additional data are available from the corresponding author, Yuya Egawa, upon reasonable request. The supplementary information includes synthetic procedures, LC-MS analyses, kinetic studies, glucose monitoring data, and analytical profiles. See DOI: https://doi.org/10.1039/d5sc02817e.
