# Supplementary material for: Fully dissolved glucose-responsive insulin delivery system based on a self-immolative insulin prodrug and glucose oxidase
Source: Chem Sci. 2025 Aug 11;16(36):16645–58. doi: 10.1039/d5sc02817e (PMC12358946; doi:10.1039/d5sc02817e)
Supplement: SC-016-D5SC02817E-s001 [file SC-016-D5SC02817E-s001.pdf]

Supplementary Information (SI) for Chemical Science.
This journal is © The Royal Society of Chemistry 2025

Supplementary Information

Fully Dissolved Glucose-Responsive Insulin Delivery System Based on a Self-Immolative Insulin Prodrug and Glucose Oxidase

Satoshi Kitaoka,[a] Minori Kojima,[a] Miho Koita,[a] Hiroki Koyama,[a] Chisato Mori,[a] Mako Okabe,[a] Ryusei Ando,[a] Kaede Kobayashi,[a] Ryo Watanabe,[a] Yuki Takano,[a] Tony D. James[b,c] and Yuya Egawa*[a]

[a] *Faculty of Pharmacy and Pharmaceutical Sciences, Josai University, 1-1 Keyakidai, Sakado, Saitama 350-0295, Japan*

[b] *Department of Chemistry, University of Bath, Bath, BA2 7AY, UK*

[c] *School of Chemistry and Chemical Engineering, Henan Normal University, Xinxiang 453007, P. R. China*

Contents

Preparation procedure for BPmoc-Ins-Asp

Scheme 1. Preparation of *p*-borono-phenylmethoxycarbonyl-modified insulin aspart (BPmoc-Ins-Asp).

Synthetic procedure for BPmoc-Gly

Scheme S2. Synthetic route for *p*-borono-phenylmethoxycarbonyl-modified glycine (BPmoc-Gly).

Fig. S1. MS spectra of peptide fragments from Ins-Asp digested with V8 protease.

Table S1. LC-MS profiles of peptide fragments from Ins-Asp digested with V8 protease.

Fig. S2. MS spectra of peptide fragments from Ins-Asp digested with V8 protease and reduced with DTT.

Table S2. LC-MS profiles of peptide fragments from Ins-Asp digested with V8 protease and reduced with DTT.

Fig. S3. MS spectra of peptide fragments from BPmoc-Ins-Asp digested with V8 protease.

Table S3. LC-MS profiles of peptide fragments from BPmoc-Ins-Asp digested with V8 protease.

Fig. S4. MS spectra of peptide fragments from BPmoc-Ins-Asp digested with V8 protease and reduced with DTT.

Table S4. LC-MS profiles of peptide fragments from BPmoc-Ins-Asp digested with V8 protease and reduced with DTT.

Fig. S5. Kinetic studies of Ins-Asp formation from BPmoc-Ins-Asp induced by H_2O_2.

Fig. S6. Glucose levels monitored after the administration of each formulation.

Fig. S7. FAB-MS spectrum of BPmoc-Gly degraded by H_2O_2.

Preparation Procedure of BPmoc-Ins-Asp

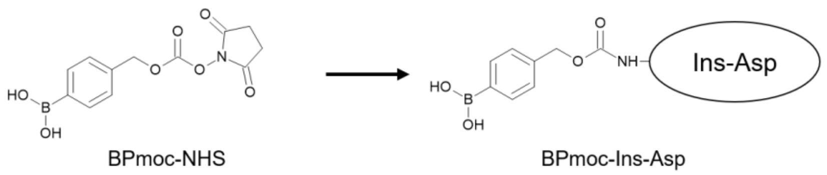

Scheme 1. Preparation of *p*-borono-phenylmethoxycarbonyl-modified insulin aspart (BPmoc-Ins-Asp).

A solution of four vials of Novorapid® Injection (100 units/mL, 10 mL) was lyophilised, and the resulting white solid containing Ins-Asp (4000 units, 140 mg, 24 μmol) was dissolved in 30 mL of dimethyl sulfoxide (DMSO, super-dehydrated) together with 150 μL of tributylamine. Separately, BPmoc-modified *N*-hydroxysuccinimide (BPmoc-NHS) (43 mg, 0.15 mmol) was dissolved in 6.0 mL of *N,N*-dimethylformamide (DMF, super-dehydrated) with 31 μL of tributylamine added. The solution containing BPmoc-NHS was then added dropwise to the Ins-Asp solution under continuous stirring under a nitrogen atmosphere at room temperature. After 3 h, 5 mL of water was added to quench the reaction. The mixture was dialysed nine times against water (1.0 L) at 4°C for at least 2 h using a dialysis tube (molecular weight cut-off 3,500 Da). The resulting solution was then lyophilised to yield BPmoc-Ins-Asp (185 mg).

The obtained BPmoc-Ins-Asp was analysed by LC-MS after enzymatic digestion, confirming that all three reactive amino groups were successfully modified with BPmoc groups. Based on this result, the theoretical molecular weight and nitrogen content of the fully modified form (bearing three BPmoc groups) were used for concentration calculations and purity evaluation, assuming that impurities do not contain nitrogen. Elemental analysis showed that the theoretical nitrogen content of the fully modified BPmoc-Ins-Asp was 14.32%, while the experimentally observed value was 10.21%, indicating an actual purity of approximately 71%.

Accordingly, all concentrations of BPmoc-Ins-Asp used in subsequent experiments (e.g., 0.10 mg/mL or 0.10 μM) were expressed as nominal values assuming full modification and 100% purity; however, the actual effective concentration should be considered to be approximately 71% of the nominal value.

Synthetic procedure of BPmoc-Gly

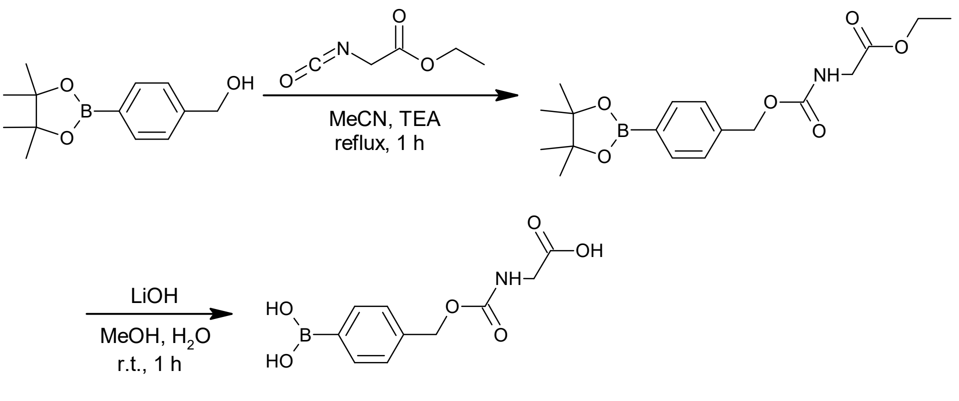

Scheme S2. Synthetic route for *p*-borono-phenylmethoxycarbonyl-modified glycine (BPmoc-Gly).

To a solution of 4-(4,4,5,5-tetramethyl-1,3,2-dioxaborolan-2-yl)benzyl alcohol (3.51 g, 15.0 mmol) and ethyl isocyanatoacetate (1.68 mL, 15.0 mmol) in acetonitrile (super-dehydrated, 150 mL), triethylamine (TEA, 4.2 mL, 30 mmol) was added. The reaction mixture was refluxed for 1 h. After cooling to room temperature, the mixture was transferred to a separatory funnel using 50 mL of ethyl acetate. The organic solution was washed twice with 1 mM hydrochloric acid (50 mL per wash) and once with brine (50 mL). The organic phase was dried over anhydrous $MgSO_4$, filtered, and concentrated under reduced pressure.

The residue containing the ethyl ester compound was dissolved in a mixture of distilled water (72 mL) and MeOH (144 mL), followed by the addition of lithium hydroxide monohydrate (3.0g, 72 mmol). The mixture was stirred at room temperature for 1 h. The reaction mixture was transferred to a separatory funnel using ethyl acetate (290 mL) and distilled water (140 mL), and the aqueous layer was washed twice with ethyl acetate (140 mL per extraction) to remove nonionic compounds from the aqueous layer. The aqueous phase was subsequently acidified to pH 1 using 1 M hydrochloric acid and extracted five times with ethyl acetate (220 mL per extraction). The combined organic layers were dried over anhydrous $MgSO_4$, filtered, and concentrated under reduced pressure. The crude product was purified by medium-pressure chromatography with a silica gel column. The elution was initiated with dichloromethane for the first 10 min, followed by a linear gradient increasing the methanol ratio to 9% over 15 min, after which the dichloromethane/methanol (91:9) ratio was maintained. The fractions containing BPmoc-Gly were collected, and their solvent was evaporated, yielding 0.965 g (3.82 mmol, 25.4% over two steps).

^{1}H-NMR (400 MHz, CD_3OD), δ 7.69 (br, 2H), 7.36 (d, 2H), 5.13 (s, 2H), 3.85 (s, 2H).

^{13}C NMR (100 MHz, CD_3OD): δ 172.21, 157.71, 138.50, 133.48, 126.39, 66.20, 41.71.

High-resolution MS (FAB, negative mode, matrix: glycerol) *m/z*: 308.0936 ($[M + \text{glycerol} - 2H_2O - H]^-$ requires 308.0947). In the MS spectrum, BPmoc-Gly was detected as a cyclic ester formed between BPmoc-Gly and glycerol.

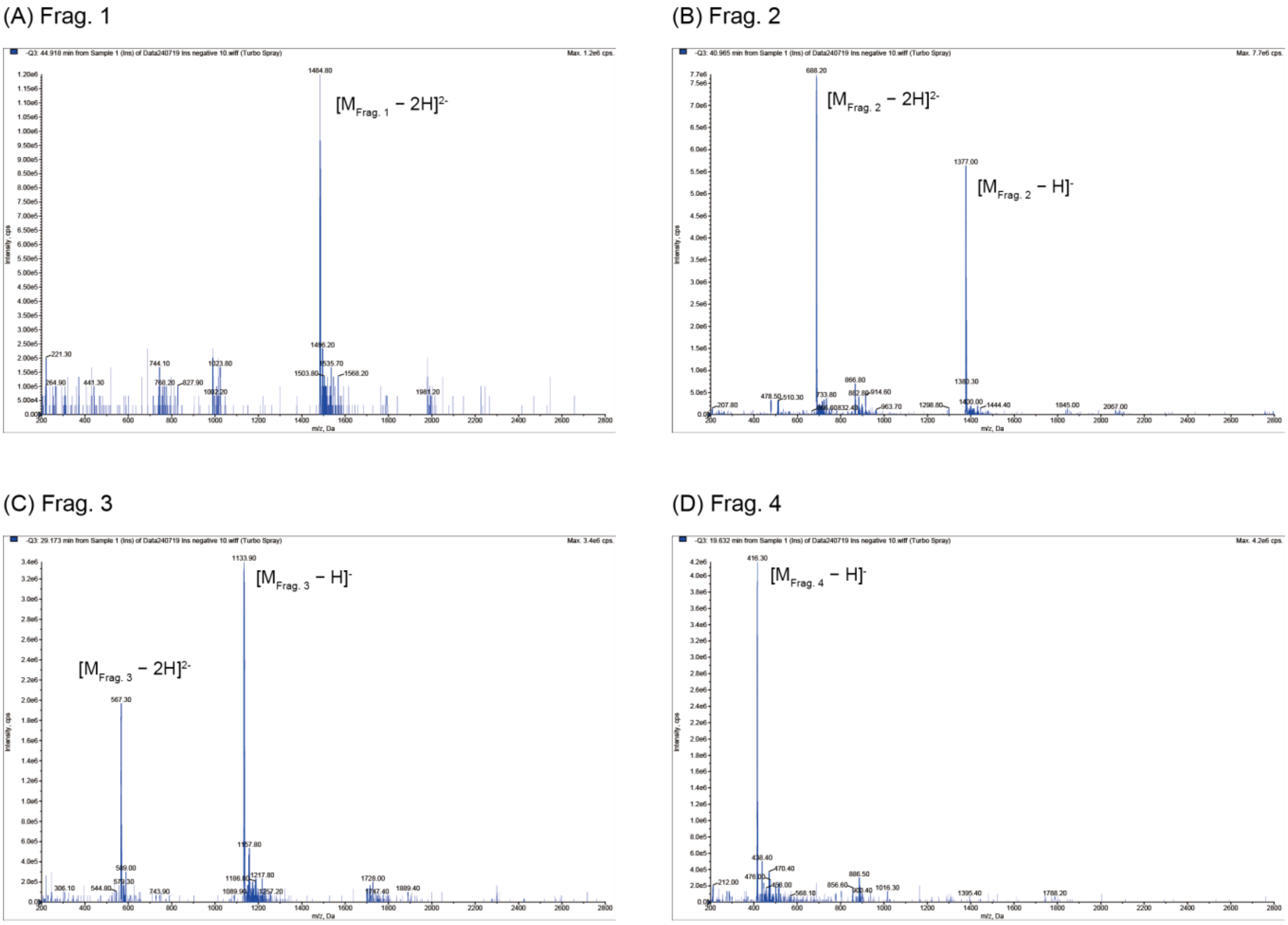

Fig. S1. MS spectra of peptide fragments from Ins-Asp digested with V8 protease. (A) Frag. 1, (B) Frag. 2, (C) Frag. 3, (D) Frag. 4.

Table S1. LC-MS profiles of peptide fragments from Ins-Asp digested with V8 protease.

Fragment	Amino acid sequence	Theoretical mass (Da)	Theoretical *m/z* for the expected ion	Observed *m/z*	Retention Time (min)
1	A-chain: QCCTSICSLYQLE B-chain: FVNQHLCGSHLVE	2971.39	1484.70 $[M_{Frag.\ 1} - 2H]^{2-}$	1484.80	45.06
2	A-chain: NYCN B-chain: ALYLVCGE	1377.55	1376.55 $[M_{Frag.\ 2} - H]^{-}$	1377.00	40.95
3	RGFFYTDKT	1134.24	1133.24 $[M_{Frag.\ 3} - H]^{-}$	1133.90	29.13
4	GIVE	416.48	415.48 $[M_{Frag.\ 4} - H]^{-}$	416.30	19.51

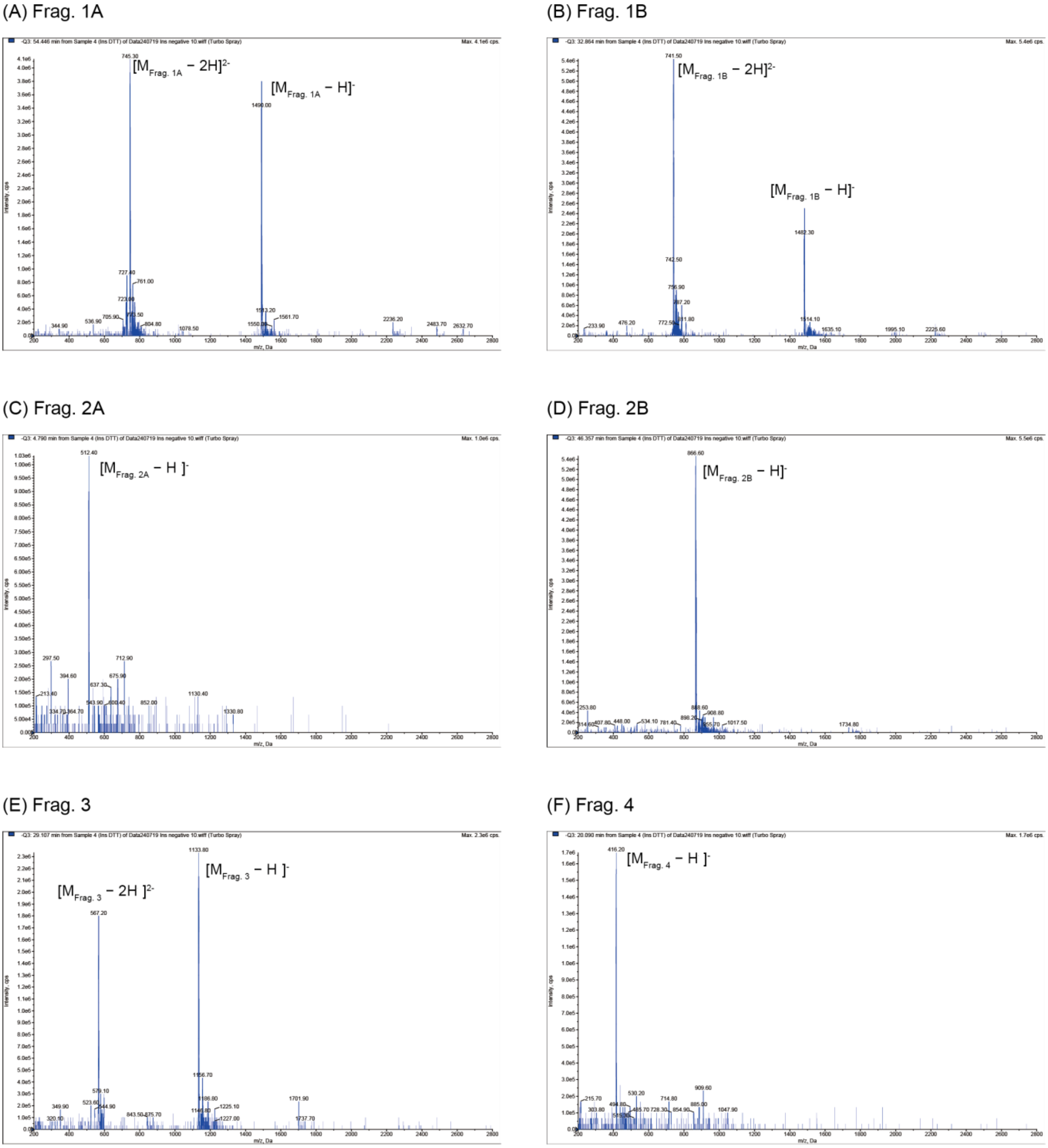

Fig. S2. MS spectra of peptide fragments from Ins-Asp digested with V8 protease and reduced with DTT. (A) Frag. 1A, (B) Frag. 1B, (C) Frag. 2A, (D) Frag. 2B, (E) Frag. 3, (F) Frag. 4.

Table S2. LC-MS profiles of peptide fragments from Ins-Asp digested with V8 protease and reduced with DTT.

Fragment	Amino acid sequence	Theoretical mass (Da)	Theoretical *m/z* for the expected ion	Observed *m/z*	Retention time (min)
1A	QCCTSICSLYQLE	1490.73	1489.73 $[M_{Frag.\ 1A} - H]^-$	1490.00	54.37
1B	FVNQHLCGSHLVE	1482.68	1481.68 $[M_{Frag.\ 1B} - H]^-$	1482.30	32.81
2A	NYCN	512.54	511.54 $[M_{Frag.\ 2A} - H]^-$	512.40	4.55
2B	ALYLVCGE	867.03	866.03 $[M_{Frag.\ 2B} - H]^-$	866.60	46.23
3	RGFFYTDKT	1134.24	1133.24 $[M_{Frag.\ 3} - H]^-$	1133.80	29.02
4	GIVE	416.48	415.48 $[M_{Frag.\ 4} - H]^-$	416.20	20.06

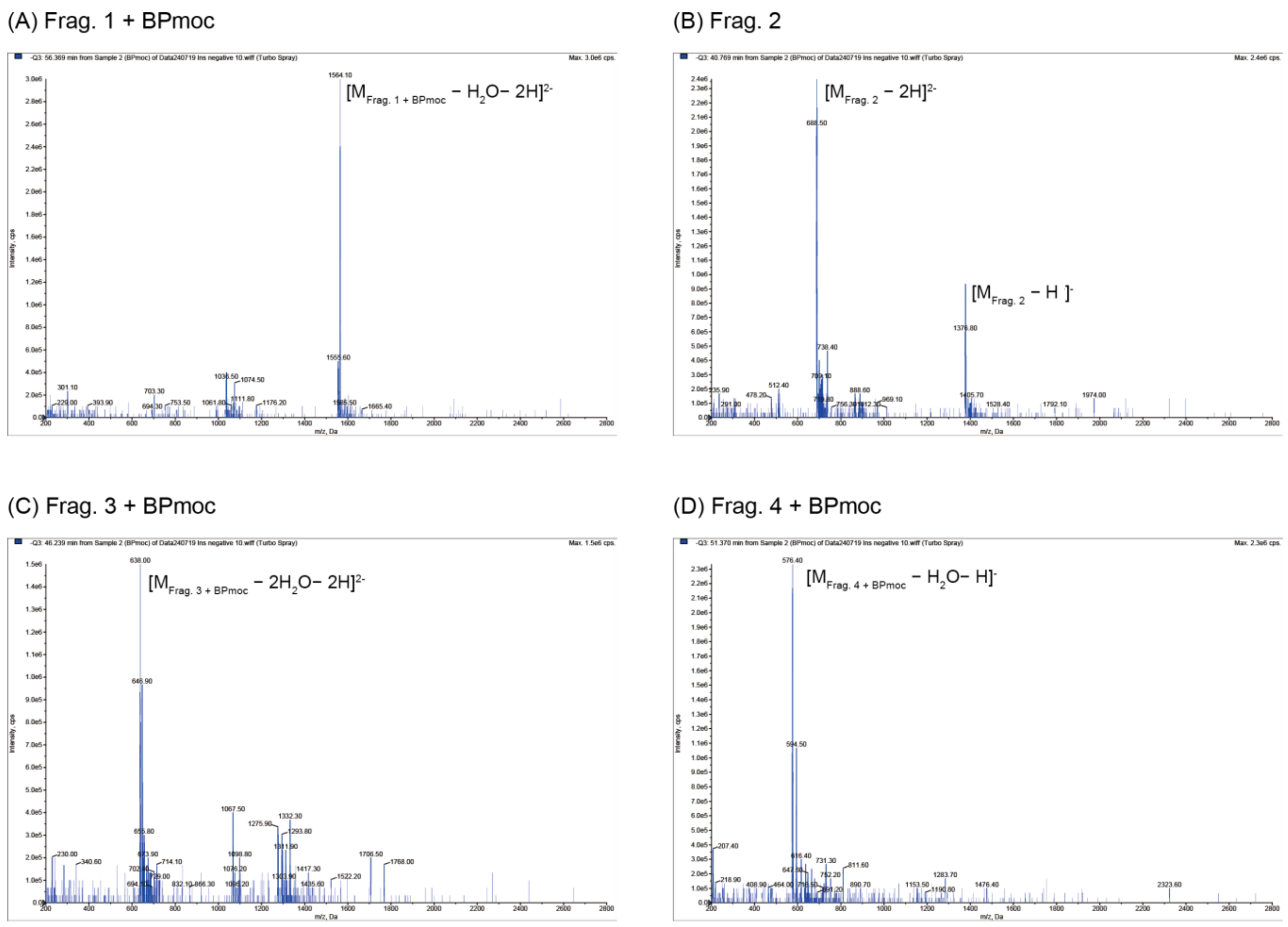

Fig. S3. MS spectra of peptide fragments from BPmoc-Ins-Asp digested with V8 protease. (A) Frag. 1 + BPmoc, (B) Frag. 2, (C) Frag. 3 + BPmoc, (D) Frag. 4 + BPmoc.

Table S3. LC-MS profiles of peptide fragments from BPmoc-Ins-Asp digested with V8 protease.

Fragment	Amino acid sequence	Theoretical mass (Da)	Theoretical *m/z* for the expected ion	Observed *m/z*	Retention Time (min)
1<br>+ BPmoc	A-chain:<br>QCCTSICSLYQLE<br>B-chain:<br>FVNQHLCGSHLVE<br>+ BPmoc	3149.34	1564.67<br>$[M_{Frag.\ 1\ +\ BPmoc} - H_2O - 2H]^{2-}$	1564.10	56.27
2	A-chain: NYCN<br>B-chain: ALYLVCGE	1377.55	1376.55<br>$[M_{Frag.\ 2} - H]^{-}$	1376.80	40.84
3<br>+ BPmoc	RGFFYTDKT<br>+ BPmoc	1312.19	637.10<br>$[M_{Frag.\ 3\ +\ BPmoc} - 2H_2O - 2H]^{2-}$	638.00	46.33
4<br>+ BPmoc	GIVE<br>+ BPmoc	594.43	575.43<br>$[M_{Frag.\ 4\ +\ BPmoc} - H_2O - H]^{-}$	576.40	51.36

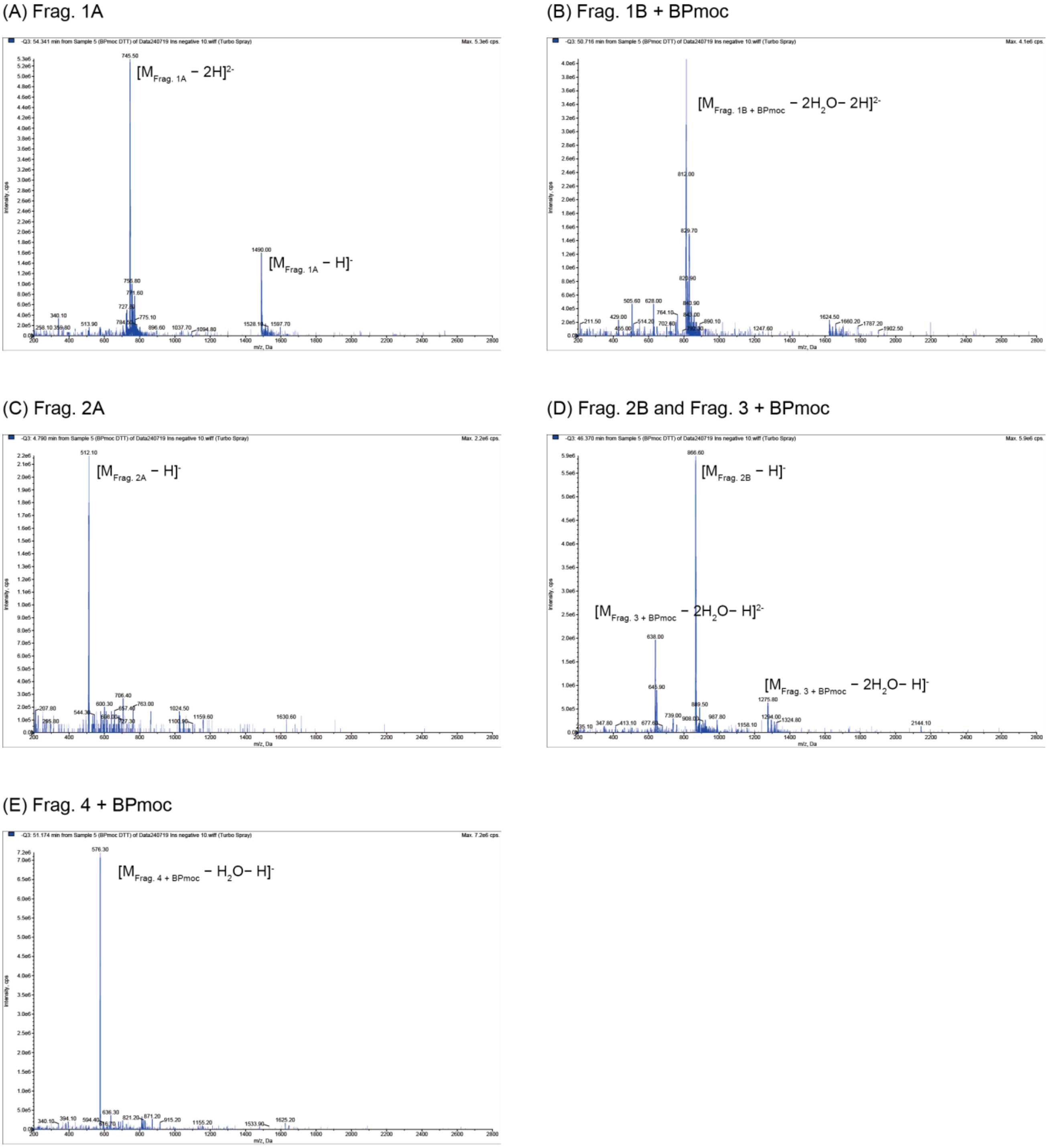

Fig. S4. MS spectra of peptide fragments from BPmoc-Ins-Asp digested with V8 protease and reduced with DTT. (A) Frag. 1A, (B) Frag. 1B + BPmoc, (C) Frag. 2A, (D) Frag. 2B and Frag. 3 + BPmoc, (E) Frag. 4 + BPmoc.

Table S4. LC-MS profiles of peptide fragments from BPmoc-Ins-Asp digested with V8 protease and reduced with DTT.

Fragment	Amino acid sequence	Theoretical mass (Da)	Theoretical *m/z* for the expected ion	Observed *m/z*	Retention time (min)
1A	QCCTSICSLYQLE	1490.73	1489.73 $[M_{Frag.\ 1A} - H]^{-}$	1490.00	54.31
1B + BPmoc	FVNQHLCGSHLVE + BPmoc	1660.63	811.32 $[M_{Frag.\ 1B + BPmoc} - 2H_2O - 2H]^{2-}$	812.00	50.67
2A	NYCN	512.54	511.54 $[M_{Frag.\ 2A} - H]^{-}$	512.10	4.85
2B	ALYLVCGE	867.03	866.03 $[M_{Frag.\ 2B} - H]^{-}$	866.60	46.39
3 + BPmoc	RGFFYTDKT + BPmoc	1312.19	1275.19 $[M_{Frag.\ 3 + BPmoc} - 2H_2O - H]^{-}$	1275.80	46.39
4 + BPmoc	GIVE + BPmoc	594.43	575.43 $[M_{Frag.\ 4 + BPmoc} - H_2O - H]^{-}$	576.30	51.32

(A)

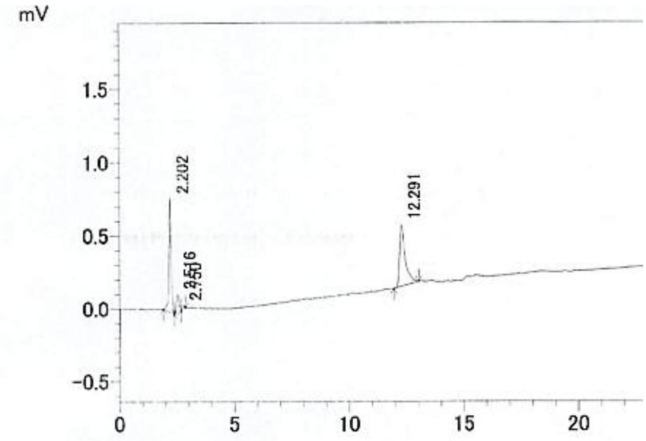

(B)

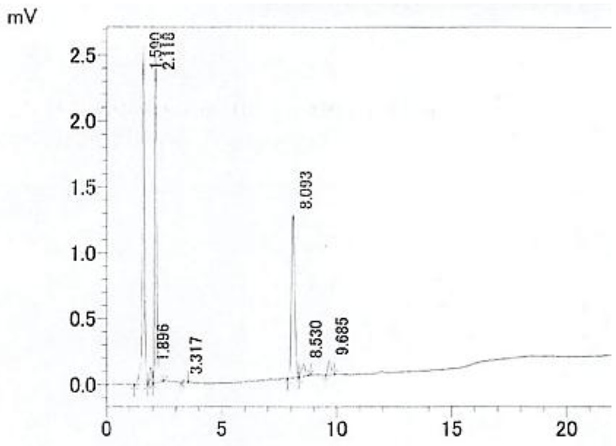

(C)

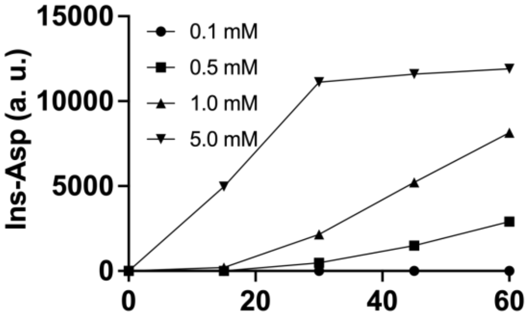

Fig. S5. Kinetic studies of Ins-Asp formation from BPmoc-Ins-Asp induced by H_2O_2. (A) HPLC chromatogram of BPmoc-Ins-Asp (0.10 mg/mL). (B) HPLC chromatogram of BPmoc-Ins-Asp (0.10 mg/mL) after 60 min of incubation at 37°C with 5.0 mM H_2O_2. (C) Ins-Asp production profiles from BPmoc-Ins-Asp (0.10 mg/mL) at different H_2O_2 concentrations (0.10, 0.50, 1.0, and 5.0 mM) at 37°C.

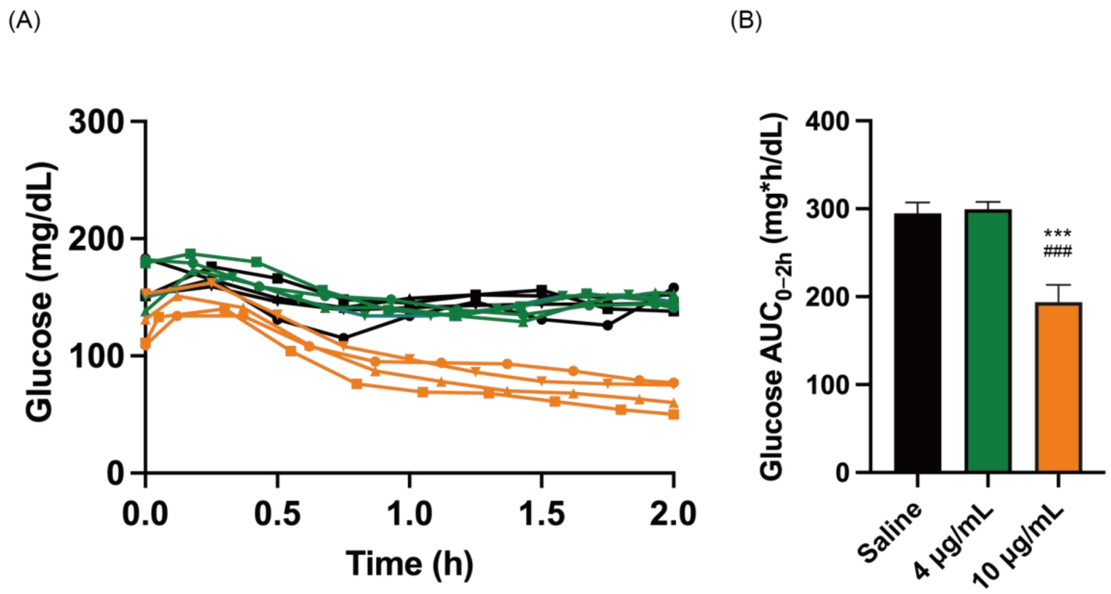

Fig. S6. Glucose levels monitored after the administration of each formulation: (Black) control (saline), (Green) 0.10 mg/mL BPmoc-Ins-Asp + 4.0 μg/mL GOx, (Orange) 0.10 mg/mL BPmoc-Ins-Asp + 10 μg/mL GOx. (A) Glucose profiles, (B) $AUC_{0–2h}$.

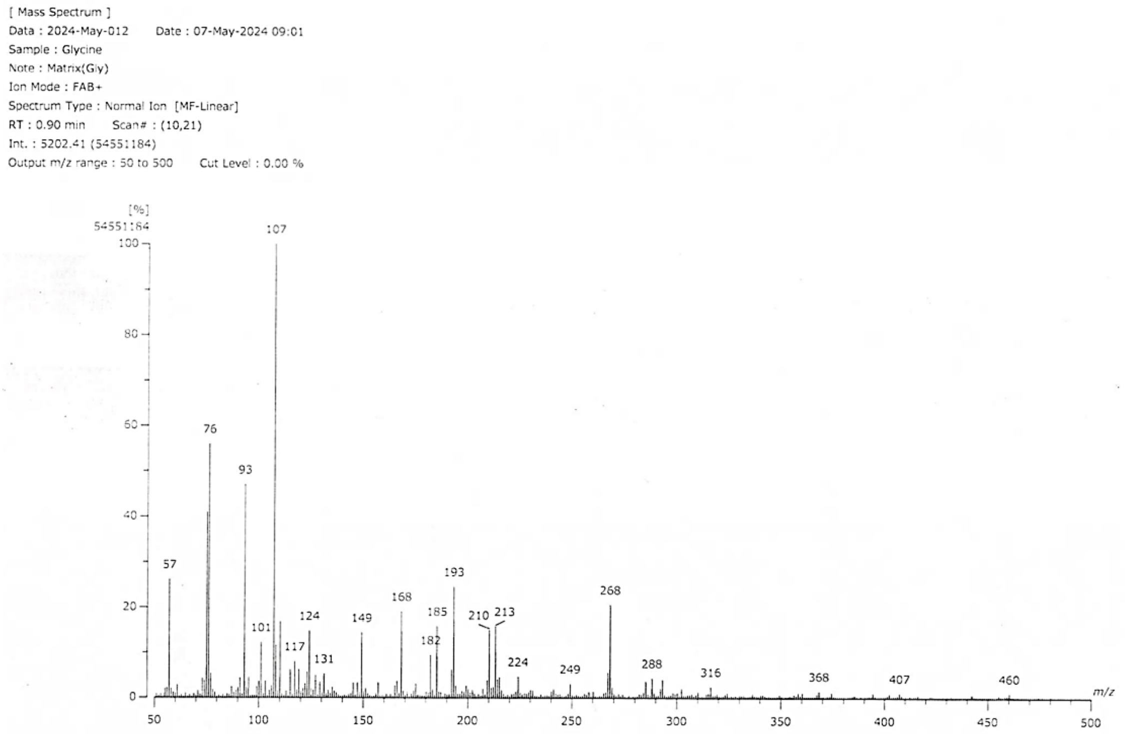

Fig. S7. FAB-MS spectrum of BPmoc-Gly degraded by H_2O_2. An aqueous solution containing 10 mM ammonium bicarbonate, 4.9 mM BPmoc-Gly and 13 mM H_2O_2 was stirred for 15 min at room temperature. The solvent was evaporated, and the residue was subjected to MS (FAB, negative mode, matrix: glycerol).
